# Barriers to Primary Care After the Affordable Care Act: A Qualitative Study of Los Angeles Safety-Net Patients’ Experiences

**DOI:** 10.1089/heq.2019.0056

**Published:** 2019-08-23

**Authors:** Sonali Saluja, Danny McCormick, Michael R. Cousineau, Janina Morrison, Lisa Shue, Kyle Joyner, Michael Hochman

**Affiliations:** ^1^ The Gehr Family Center for Health Systems Science and Innovation, Department of Medicine, Keck School of Medicine, University of Southern California, Los Angeles, California.; ^2^ Cambridge Health Alliance, Harvard Medical School, Cambridge, Massachusetts.; ^3^ Department of Preventive Medicine, Keck School of Medicine, University of Southern California, Los Angeles, California.; ^4^ The Wellness Center, Los Angeles, California.; ^5^ Department of Public Health, Los Angeles County, Los Angeles, California.; ^6^ Keck School of Medicine, University of Southern California, Los Angeles, California.

**Keywords:** access to care, vulnerable populations, health care reform, insurance, primary care

## Abstract

**Purpose:** Millions of people gained health care coverage in Los Angeles after the Affordable Care Act (ACA); however, challenges with obtaining and utilizing primary care still persist, particularly in the safety net. In this study, we explore barriers to accessing primary care services among safety-net patients in Los Angeles after Medicaid expansion and implementation of other programs for safety-net patients after the ACA.

**Methods:** We conducted qualitative interviews, in Spanish and English, with 34 nonelderly adult patients in 1 of 3 insurance groups: Medicaid, MyHealthLA (a health care program for low-income undocumented individuals), or uninsured. We recruited participants from three sites in Los Angeles in 2017. We analyzed our interviews using a framework approach and included emerging concepts from participant responses.

**Results:** We identified seven themes regarding barriers to accessing primary care: understanding the concept of primary care, finding a primary care provider (PCP), switching PCPs, getting timely appointments, geography and transportation, perceived cost or coverage barriers, and preferring emergency or urgent care over primary care. Patients with Medicaid were more likely to report barriers compared with other groups. Uninsured patients were less likely to understand the concept of primary care. Patients with MyHealthLA noted getting timely appointments and cost of care to be significant barriers.

**Conclusion:** Despite Medicaid and other coverage expansions for safety-net patients after the ACA, substantial barriers to accessing primary care persist. Addressing such barriers through the development of targeted interventions or broader policy solutions could improve access to primary care for safety-net patients in Los Angeles.

## Introduction

The Affordable Care Act (ACA) expanded health insurance to millions of Americans in 2014, including ∼1.7 million people in Los Angeles County.^1^ The vast majority of those newly covered under the ACA in Los Angeles County gained Medicaid (called Medi-Cal in California).^1^ In addition, to provide health care for the large number of undocumented people in Los Angeles, the County created the MyHealthLA health care program, which was implemented at the same time as the ACA with the goal of providing access to primary care at community clinics.^2^ Thus, the ACA has brought sweeping changes to Los Angeles County's health care landscape, particularly for individuals within the safety net.

Despite major gains in insurance coverage across the country following implementation of the ACA, barriers to accessing primary care services persist, particularly in the safety net.^3^ Nationally, gains in the percentage of people with primary care providers (PCPs) after the ACA were observed in the privately insured but not among those with Medicaid.^4^ In California, patients with Medi-Cal had a harder time accessing primary compared with those with employer-sponsored coverage, and these disparities did not improve after the ACA; furthermore, low-income people with Medi-Cal were twice as likely to be refused new patient appointments in primary care 1 year after the ACA.^5^ In the most recent and comprehensive study of access to care for Los Angeles County residents conducted after the ACA, nearly a quarter of those surveyed reported at least some difficulty obtaining care when they needed it; this proportion was >40% in the poorest income group (those making <100% of the federal poverty level).^6^

Several factors in Los Angeles may contribute to challenges patients experience in accessing needed care in the post-ACA era. First, only slightly more than half of providers in California accept new Medi-Cal patients, one of the lowest rates of Medicaid acceptance in the country.^7^ Second, California, and Los Angeles in particular, has a high proportion of patients enrolled in Medi-Cal-managed care plans, which typically offer narrow provider networks.^8,9^ Finally, Los Angeles has a relatively high percentage of racial and ethnic minorities, non-English speakers, undocumented persons, and persons living below the federal poverty level—there is evidence that such groups face greater barriers to accessing health care, including primary care.^10,11^ Other metropolitan areas across the nation are experiencing similar demographic shifts, including growth in populations who are foreign born, racial/ethnically diverse and enrolled in Medicaid-managed care, along with a limited supply of PCPs.^12–15^

The MyHealthLA program was designed to improve access to health care for undocumented people, a group that has now the highest uninsurance rate in the state.^16^ The program provides a primary care medical home at one of >200 community clinics in the County for individuals who, based on their income (<138% of the federal poverty level), would qualify for Medi-Cal, but due to their immigration status, are ineligible.^17^ However, unlike insurance, the program has no portability and coverage for specialty services, or tertiary care is only available at County medical centers.

While coverage expansion has been observed throughout the County, little is known about the experiences of safety-net patients in obtaining primary care services after the ACA, or how these experiences may differ by insurance group. In this study, we conduct qualitative interviews with safety-net patients from multiple health systems to understand current barriers to accessing and using primary care in Los Angeles.

## Methods

### Interview guide and data collection instrument

We evaluated barriers to primary care services through interviews with patients using both open-ended and focused questions. We developed our interview guides based on the existing literature and a previous study done in Massachusetts on safety-net patients' access to care.^18^ All authors, as well as insurance enrollment experts and patient navigators, reviewed the guide and suggested changes to optimize question understandability. Our definition of primary care was based on Barbara Starfield's definition: care that is first contact, accessible, longitudinal, and comprehensive as well as in the ambulatory setting.^19^ We specifically asked participants if they could identify a regular primary care clinic (PCC) where they could go for nonemergency care and also a single individual who served as their PCP. We used different terms or examples to describe a PCC if needed. In addition to asking open-ended questions, we asked specifically about established barriers to primary care services, such as geography, cost, insurance acceptance, conceptual knowledge, and awareness. Guides were translated into Spanish and then back translated for accuracy. We pilot tested the interview guides in both English and Spanish, and made iterative modifications to improve question comprehension and flow. The first author and three trained research assistants conducted all interviews; two interviewers who were proficient in Spanish conducted interviews with Spanish-speaking patients.

### Sample selection and recruitment

We recruited participants during the summer of 2017 from three sites in East Los Angeles neighborhoods: The Los Angeles County Hospital (LAC+USC), a publicly funded hospital; The Wellness Center, a social service resource center for safety-net patients; and White Memorial Medical Center, a private safety-net hospital in East Los Angeles. We recruited most of our participants from the urgent care (UC) or emergency department (ED) waiting rooms of the two hospitals using a convenience sampling approach. We purposefully sought to recruit patients from these settings to better understand the experiences of patients with unmet needs, who may be struggling to access primary care. Patients with any of the following three safety-net insurance/health care coverage groups were eligible: Medi-Cal, MyHealthLA, and those without any coverage (uninsured patients including those with only emergency Medi-Cal). We included nonelderly adults (ages 18–64), the target population of the coverage expansions under the ACA, and excluded patients who were non-English or non-Spanish speaking or were too sick to be interviewed.

We approached all potential participants to discuss the study, provided them with study materials to review, and obtained informed consent from them. Because of a paucity of patients with MyHealthLA at our study sites, we augmented their recruitment through phone calls to people who had enrolled in the program within the last year. We audio-recorded and transcribed all interviews when permitted (two participants requested not to be recorded) and took field notes. Participants received a US$25 debit card as compensation for their time. This study was approved by the University of Southern California Health Science's Institutional Review Board.

### Data analysis

We used a mixed inductive and deductive approach to analyze the qualitative data. First, four coders, two physicians with experience and training in qualitative coding, and two trained research assistants independently identified key codes. We derived deductive codes from the previous literature describing barriers to ambulatory care as well as prespecified codes based on knowledge of the health care system. Inductive codes emerged from our transcripts and discussions of the interviews. Through an iterative process, we developed a coherent set of codes (∼2 researchers coded a sample of 10 transcripts). We also gathered quantitative data on discrete variables, such as participant demographics and self-reported health care utilization, and calculated descriptive statistics. We organized our qualitative data using a framework approach, which has been previously used in health care and policy-related qualitative research.^20^ We used Atlas TI software (Version 1.6.0) to assist with qualitative coding and Excel to create a framework matrix. We then analyzed our matrix for patterns and to generate hypotheses relevant to our research questions. Specifically, we examined differences in themes by insurance/health care coverage group and other health system characteristics.

## Results

The majority of study participants were childless adults and had an annual income <138% of the federal poverty level (Table 1). Over 40% of our participants were Spanish speaking only, reflecting the relatively high percentage of Latino individuals served by the safety net in Los Angeles.

**Table 1.** Characteristics of Participants

Characteristics (*N*=34)	*N*/mean (% of participants/SD)
Insurance group	
Medi-Cal	20 (58.8%)
Uninsured	7 (20.6%)
MyHealthLA	7 (20.6%)
Mean income	US$12,873 ($9,876)
Mean household size	2.7 (1.8)
Mean No. of dependents	0.8 (1.2)
Spanish speaking only	14 (41.2%)

SD, standard deviation.

About one-third of the participants did not have a regular PCC (Table 2); however, this varied substantially by insurance type and status: only 28% of uninsured participants had a regular PCC compared with 70.0% of Medi-Cal participants and 85.7% of MyHealthLA participants. About 18.2% of participants with a regular PCC still used UC or the ED at least on some occasions for their primary care needs; this percentage was much higher (58.3%) among participants without regular primary care. Participants with regular primary care tended to report more chronic medical problems and slightly more visits to the ED and UC, but reported a similar number of hospitalizations.

**Table 2.** Characteristics and Self-Reported Health Care Utilization of Participants With and Without a Regular Primary Care Clinic

	Participants with a regular PCC (*N*=22)	Participants without a regular PCC (*N*=12)
Uninsured	9.1% (2)	41.7% (5)
Insured	90.9% (20)	58.3% (7)
Medi-Cal	63.6% (14)	50.0% (6)
MHLA	27.2% (6)	8.3% (1)
Enrollment site^a^		
Clinic	20.0% (4)	14.3% (1)
Hospital	25.0% (5)	42.9% (3)
Department of Public and Social Services	35.0% (7)	42.9% (3)
The Wellness Center	15.0% (3)	0 (0%)
Unknown	5.0% (1)	0 (0%)
Has a PCP	72.7% (16)	N/A
Uses UC for primary care	18.2% (4)^b^	58.3% (7)
Two or more chronic conditions	77.3% (17)	41.7% (5)
Homeless	13.6% (3)	0 (0%)
Average No. of ED/UC visits in the last 3 years	7.3	5.5
Average No. of hospitalizations in the last 3 years	0.7	0.7
Avoided care for any reason in the last 3 years	40.1% (9)	41.7% (5)

^a^ Denominators for these sites only include participants with Med-Cal or MyHealthLA.

^b^ Three of these participants reported using UC sometimes for their primary care needs; one participant used UC consistently for his/her primary care needs.

ED, emergency department; PCC, primary care clinic; PCP, primary care provider; UC, urgent care.

We identified seven specific themes related to barriers to accessing and using primary care (Tables 3 and 4).

**Table 3.** Percentage of Study Participants Identifying Specific Themes Around Barriers to Primary Care

Themes	Examples of barriers	Total (*N*=34)	Medi-Cal (*N*=20)	MyHealthLA (*N*=7)	Uninsured (*N*=7)
Understanding primary care	Unfamiliar with the concept of primary care	14.7% (5)	15.0% (3)	0% (0)	28.6% (2)
	Place low value in primary care				
Finding a PCP or PCC	Unfamiliar with PCP/clinic options	23.5% (8)	30.0% (6)	28.6% (2)	0% (0)
	PCP/clinic was chosen for them, not by them				
	Unaware they had a PCP				
Switching PCPs or PCCs	Unaware of how to change PCPs	14.7% (5)	25.0% (5)	0% (0)	0% (0)
	Process of switching is cumbersome				
Wait times for primary care appointments	Long wait times till the next appointment	29.4% (10)	30.0% (6)	42.9% (3)	14.2% (1)
	No walk-in visits				
	Long phone wait times				
	Clinic hours conflict with work				
Location and transportation	Clinic is too far from home	17.6% (6)	30.0% (6)	0% (0)	0% (0)
	Difficulty using/acquiring transportation				
Using ED/UC as primary care	ED/UC was faster or cheaper than primary care	32.4% (11)	35.0% (7)	28.6% (2)	28.6% (2)
	ED/UC was familiar				
Cost or coverage	Actual cost or coverage barriers	17.6% (6)	5.0% (1)	57.1% (4)	14.2% (1)
	Misconceptions about cost or coverage				

**Table 4.** Themes and Representative Quotes on Barriers to Primary Care

Themes	Representative quote
Understanding primary care	“The only primary doctor that I remember I've ever had was in neurology.”—uninsured patient
	“I use urgent care … when I get a cough, or when I need refills with my medicine.”—uninsured patient
Finding a PCP or PCC	“I didn't choose but just because I didn't reply to the letter that I received, they chose [my PCP] for me. That's what I didn't like.”—Medi-Cal patient
	“It would've been nice to at least know where [my clinic was] until I needed it and then find out it's a two-hour bus ride. I don't want to do that every time I need to see a doctor.”—Medi-Cal patient
Switching PCPs or PCCs	“But when it came down to choosing [a PCP] for this hospital specifically, it was a struggle, because either they didn't accept my [managed care plan], or it was just gonna be a male doctor and I wanted to be seen by a female doctor.”—Medi-Cal patient
	“I would literally have to leave my house, come here, talk to … member services, or I can go to [the hospital] and see if my nurse there will help me find a new doctor.”—Medi-Cal patient
Wait times for primary care appointments	“I will call and sometimes [my doctor] is booked all month so I will have to wait for the next month.”—MyHealthLA patient
	“I've tried walking in, but they said that they stopped doing walk in visits two year ago.”—Medi-Cal patient
Location and transportation	“I'm trying to get my own place and a lot of things could happen when I'm [bringing my] medicine on the bus. If anything went wrong … because a lot of stuff has happened to me in the past.”—Medi-Cal patient
Using ED/UC as primary care	“I don't go over [to that clinic]. Believe it or not, here in the hospital they're faster. Urgent care, it's fast.”—Medi-Cal patient
	“Sometimes when I do try to go [to the clinic] it's always, ‘Wow. We can't accept walk-ins because the person's so busy today’ … so I just go to the emergency room.”—Medi-Cal patient
	“I had all these problems that came up a month ago: the afib … the COPD, the kidney problems, and I spent about 10 days [in the hospital]. They put me on a bunch of medications and released me, so here I am trying to get aftercare.”—Medi-Cal patient
Cost or coverage	“When I went to the free clinic with the primary doctor, she suggested I [get an ultrasound] but there they'd charge me 75 dollars and … I couldn't pay it. But my friend told me, why are you going to pay since you have the emergency Medi-Cal, so go to the hospital.”—MyHealthLA patient
	“It doesn't cover much. If you have a dental pain or one of your teeth molars, the only thing is ‘Well, we'll extract it.”—MyHealthLA patient

### Understanding or valuing primary care

Many uninsured participants and those with Medi-Cal were not aware of the concept of primary care and did not see value in having a PCP. However, all the participants with MyHealthLA were familiar with the concept. Two participants regarded UC as their PCC and did not seem to be aware of alternatives to UC. Some participants did not think they would benefit from primary care. For example, two participants thought that primary care was for “sicker” people and impractical for healthy people.

### Finding a PCC or PCP

Nearly a quarter of participants reported difficulty in finding or selecting a PCC or PCP. This barrier affected participants with both Medi-Cal and MyHealthLA. Uninsured patients were much less likely to have a regular PCC or PCP; however, most did not report that they were trying to find one. One specific challenge participants reported was a lack of guidance with selecting a PCP. Participants were unaware of their options or did not understand the network limitations of their managed care plans. Some participants with Medi-Cal were frustrated with being assigned a PCP by their managed care plan. Of the nine participants who were assigned to a PCP by their managed care plan, three had never visited their assigned PCPs and instead used UC for their primary care needs. Some participants also described facilitators that helped them find a PCP—these included family members, friends, and navigators who helped them enroll in insurance.

### Switching PCCs or PCPs

Five participants, all of whom had Medi-Cal, also struggled with switching their PCCs or PCPs. Most lacked information about how to initiate the process of switching. Reasons for wanting to change PCCs or PCPs included the clinic distance, preference for a different PCC, health system or PCP, resources available at another PCC (such as laboratories, imaging, or extended hours), and dissatisfaction with their current PCP. One participant, who was assigned to a managed care plan that was not accepted by her preferred PCP, found the process of switching her managed care plan (to the one accepted by her preferred PCP) to be cumbersome.

### Wait times and availability for PCC appointments

Over a fourth of the participants, primarily those with Medi-Cal or MyHealthLA, reported challenges with wait times and appointment availability in accessing primary care. The most common issues around wait times was prolonged wait times for a PCC appointment (seven participants waited >2 weeks). This was followed by other challenges, including no option for walk-in appointments, waiting for several hours in the clinic before appointments, and being placed on hold on the phone (sometimes for hours) when attempting to reach PCC staff. One participant used the UC for her primary care needs because her PCC was not open during her nonwork hours. In contrast, another participant shared that her PCC recently expanded its hours, allowing her to book appointments after work instead of going to UC.

### Location and transportation barriers

Participants with Medi-Cal were the only group to cite transportation or location as a significant barrier to accessing primary care. Most participants used the bus to get to their PCC or UC/ED, though some walked or used personal transportation. Location of the PCC was generally cited as a barrier if it took too long to get to the clinic or if the clinic was in an unfamiliar area. One participant, who had previously been assaulted on a bus, reported concerns for his safety when using public transportation. Most participants who reported having a regular PCC only had a short walk or bus ride to their clinic.

### Perceived cost or coverage barriers

Cost barriers to primary care were commonly reported among those with MyHealthLA (the majority reported a barrier). For patients with MyHealthLA, visits to designated primary care sites are free; however, coverage of imaging, laboratories, and specialty care are restricted to certain County sites. Several participants noted that imaging studies were too costly for them when ordered by their PCC. Some MyHealthLA participants incorrectly assumed that there were costs associated with all outpatient services such as specialty care (which is free through the County health system). Two participants (one with Medi-Cal and the other with MyHealthLA) were frustrated with inadequate dental coverage. One uninsured participant assumed that she could not afford to pay for primary care visits anywhere without insurance.

### Preference for urgent or emergency care

Participants from all insurance groups, particularly those without a regular PCC, perceived several advantages to receiving care in UC or the ED. A few participants thought that their wait times in UC or the ED were shorter than what they had experienced in primary care. Participants who did not have PCC with walk-in appointments appreciated that the ED or UC never refused to see them. Several participants expressed their satisfaction with the care they received in the ED, UC, or both, and thus continued to use them for primary care needs. Participants also visited UC because it was a familiar place for them and often close to or attached to the hospital where they received care.

## Discussion

Three years after the ACA, our study suggests that access to primary care is still challenging for some patients in the safety net in Los Angeles County. We found seven key themes that described barriers participants face in accessing primary care. Our analysis also suggests that these barriers differentially impact patients based on their health care coverage group, with Medi-Cal recipients reporting the most challenges to accessing primary care services.

Participants without insurance were more likely to have difficulty understanding the concept of primary care, and preferred using the UC or ED for their primary care needs. While uninsured participants were much less likely to report other barriers noted in our study, it is important to note that these participants were often not trying to access primary care.

MyHealthLA participants seemed to report fewer barriers overall to accessing primary care. This is consistent with the program's strategy of enrolling patients predominantly through community PCCs and their strong emphasis on primary care.^21^ However, long appointment wait times were problematic for MyHealthLA participants, a barrier reported across all insurance groups. MyHealthLA participants were also more likely to report cost barriers to care due to limited outpatient coverage of laboratories and imaging tests.^22^ In fact, some participants sought primary care in the Los Angeles County ED, where such services are covered by MyHealthLA.

Participants with Medi-Cal experienced all seven barriers that we identified, though only one person reported a cost barrier to care. Unlike patients in the MyHealthLA program, which provides instant enrollment and assignment to a PCC, Medi-Cal recipients can experience a confusing and prolonged enrollment process, which includes selecting a managed care plan before selecting an in-network PCP.^8,23^ Challenges with a burdensome enrollment process may be compounded by low Medi-Cal acceptance rates among providers and limited in-network providers within managed care plans.^24^ Patients with Medi-Cal also reported a preference for receiving care in the ED or UC. Factors such as social instability and distrust of the health care system may contribute substantially to this theme, though systemic problems may also play a role.^25,26^

Our study has several unique strengths and limitations. We recruited participants from multiple sites, including both private and public health systems. We verified insurance status for participants through the hospital registration systems, rather than using the less accurate method of self-reporting.^27^ We included homeless patients and patients with mental illness—groups that are often overlooked in health policy studies. Recruitment sites were geographically limited to East Los Angeles, and we only interviewed Spanish- and English-speaking participants, reducing the generalizability of our study. Although language was not reported to be a significant barrier to accessing primary care for most of the participants, language barriers are likely to be more problematic in non-Spanish- and non-English-speaking populations in Los Angeles. Finally, in recruiting a convenience sample of patients, we may have biased our sample to include participants with either very positive or very negative experiences with the health care system. However, given that this is a qualitative analysis with the goal of generating hypothesis, quantifying the magnitude of barriers patients' experienced was beyond the scope of this study.

Our interviews revealed notable barriers to accessing primary care that could be targets for both tested and novel interventions or policy solutions. First, efforts to educate patients on the benefits of establishing with primary care may be worthwhile; however, such initiatives should be adequately resourced as safety-net primary care systems are often difficult for patients to navigate. Second, at a policy level, broadening Medicaid-managed care networks and increasing reimbursements for PCPs could ameliorate some of these systemic barriers to care. Third, addressing administrative challenges within Medi-Cal, such as reducing the enrollment time and allowing enrollees to select a primary care site during enrollment, may improve primary care utilization. Fourth, health systems should consider innovations in transportation and telemedicine, which may make care more accessible for certain populations. Finally, health systems should invest in navigation services that help vulnerable patients enroll in health care coverage and find PCPs that fit their needs.

## Conclusion

In Los Angeles, enthusiastic adoption of the ACA, and additional programs providing health care for undocumented people, opened the door to health care for millions. While these changes are a critical first step, additional steps are needed to ensure meaningful health care access for all. Ultimately, targeted interventions and comprehensive policy solutions could lead to improved primary care access and utilization.

## Abbreviations Used

ACA: Affordable Care Act
ED: emergency department
PCC: primary care clinic
PCP: primary care provider
UC: urgent care
